# Bioinformatics Analysis of the Anti-Inflammatory Mechanism and Potential Therapeutic Efficacy of *Kezimuke granules* in Treating Urinary Tract Infections by Inhibiting NLRP3 Inflammasome Activation

**DOI:** 10.3390/ijms26041764

**Published:** 2025-02-19

**Authors:** Alhar Baishan, Alifeiye Aikebaier, Dilihuma Dilimulati, Nuerbiye Nueraihemaiti, Yipaerguli Paerhati, Sendaer Hailati, Nulibiya Maihemuti, Wenting Zhou

**Affiliations:** 1 Department of Pharmacology, School of Pharmacy, Xinjiang Medical University, Urumqi 830017, China; alhar@stu.xjmu.edu.cn (A.B.); alifeiye@stu.xjmu.edu.cn (A.A.); dilihuma@stu.xjmu.edu.cn (D.D.); nurbiye@stu.xjmu.edu.cn (N.N.); yipaerguli@stu.xjmu.edu.cn (Y.P.); sendaer@stu.xjmu.edu.cn (S.H.); nurbiye24@stu.xjmu.edu.cn (N.M.); 2 Xinjiang Key Laboratory of Natural Medicines Active Components and Drug Release Technology, Urumqi 830017, China; 3 Xinjiang Key Laboratory of Biopharmaceuticals and Medical Devices, Urumqi 830017, China; 4 Engineering Research Center of Xinjiang and Central Asian Medicine Resources, Ministry of Education, Urumqi 830017, China

**Keywords:** bioinformatics, NLRP3 inflammasome, machine learning, molecular docking, UHPLC-MS/MS

## Abstract

*Kezimuke granules* (KZMK), derived from traditional Kazakh folk medicine, exhibit a variety of pharmacological properties. Long-term clinical studies have demonstrated their efficacy in clearing heat, detoxifying, promoting qi circulation, and alleviating gonorrhea. However, their specific pharmacological effects on urinary tract infections remain unclear. This study employed UHPLC-MS/MS technology to identify the blood components of KZMK and integrated network pharmacology with bioinformatics analysis for molecular docking validation. The anti-inflammatory activity of KZMK was further evaluated using a rat model of LPS-induced cystitis. A total of 17 components in KZMK were identified as capable of entering the bloodstream. Predictive analysis revealed that its primary targets include Caspase-1, NLRP3, STAT1, TLR4, and TNF, with the NLRP3 inflammasome signaling pathway emerging as the key mechanism. In vivo studies showed that KZMK effectively reduced the white blood cell (WBC) count and bladder index in urine sediments of rats with cystitis. Additionally, KZMK alleviated bladder congestion, edema, and histopathological changes in the animals. Treatment with KZMK led to decreased levels of IL-18 and IL-1β cytokines. KZMK significantly inhibited the expression of NLRP3, GSDMD, and Caspase-1 in LPS-induced cystitis, further confirming its anti-inflammatory effects. These findings indicate that KZMK provides protection against LPS-induced cystitis, primarily by inhibiting the activation of the NLRP3 inflammasome. Collectively, the results suggest that KZMK holds promise as a potential therapeutic option for urinary tract infections.

## 1. Introduction

Urinary tract infections (UTIs) rank among the primary prevalent infectious diseases globally, impacting approximately 130 to 175 million individuals each year. They are the second most common type of infection, surpassed only by respiratory tract infections [1]. In China, the incidence of UTIs is approximately 2%, with women being particularly susceptible; approximately 60% of women encounter at least one UTI throughout their lives [2]. UTIs encompass both acute and chronic inflammatory conditions impacting the urinary tract mucosa or tissue. These infections originate from the invasion and multiplication of pathogens within the affected areas [3]. According to the site of infection, urinary tract infections can be divided into two primary categories: infections of the upper urinary tract, which encompass pyelonephritis and ureteritis, and infections of the lower urinary tract, consisting of urethritis and cystitis [4]. The main clinical manifestations are urination frequency, urgency, urination pain, suprapubic bladder area or perineal discomfort, and urethral burning sensation [5].

UTIs are most frequently induced by urinary tract-specific Escherichia coli (UTEC), which serves as the primary causative agent. Roughly 80 percent of all UTIs can be ascribed to UTEC [6,7]. UTEC produces specific surface proteins known as adhesins, which facilitate their adherence and penetration of the epithelial cells lining the bladder. If the infection persists in the bladder, it is classified as a simple urinary tract infection. However, if the infection is not effectively eradicated, some strains of UPEC may ascend to the ureters and kidneys, leading to more severe infections termed complicated UTIs. These infections can result in renal damage and, in severe cases, renal failure. Complicated UTIs caused by UPEC account for a significant proportion of bacterial infections acquired in hospitals, ranging from 40% to 50%. These infections result in heightened morbidity rates, extended durations of hospitalization, and a considerable financial strain on healthcare systems [8]. In recent years, especially during empirical treatment, the overuse of antimicrobial agents has changed UTI pathogen distribution and led to drug resistance. The sharp rise in multi—resistant bacteria (MDR, resistant to three or more drugs) in UTIs poses challenges to clinical anti—infective treatment [9]. Resistance was significantly linked to the use of antimicrobial agents, especially fluoroquinolones and antifalse—positive penicillins. Prolonged pre—UTI use of any agent was also significantly associated with resistance [10]. When considering the issue of resistance, it is evident that bacteria exhibit widespread antibiotic resistance primarily resulting from the generation of multiple toxins produced by Gram-negative bacteria [11]. Therefore, it is necessary to fully utilize the advantages of TCM and ethnomedicine in research, combined with the research methods of basic biology, in order to find more UTI therapeutic drugs to meet clinical needs. This is to achieve the rational use of antibiotics to avoid the adverse consequences of their abuse.

*Kezimuke* granule (KZMK)is the traditional medicine prescription of the Xinjiang Kazak nation, which is composed of three kinds of medicinal materials, such as *Equisetum hiemale* L., *Helichrgsam arenarium* (L.)Moeneh. and *Vaccinium myriillis* L. *Vaccinium myrtillus* is the dried leaves and fruit of the genus *Vaccinium* in the family *Rhododendron*. T *Helichrysum arenium* (L.) *Moech* is the dry aboveground part of the *Chrysanthemum genus* in the Asteraceae family. *Equisetum hiemale* L. is a plant species belonging to the *Equisetum* family and the *Equisetum* genus [12].

**Figure 1:**
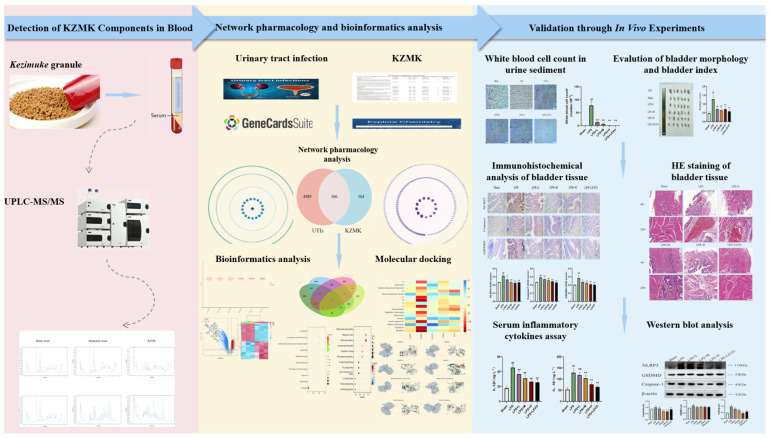
Research Flowchart for the Therapeutic Effect of KZMK Granules on UTIs. In this paper, we employed UPLC-MS/MS to detect the blood components of KZMK granules, identifying a total of 17 blood components as potential active compounds and targets. Subsequently, we collected target information for these components using the SwissTarget Prediction database. Meanwhile, we obtained targets related to UTIs from the GeneCards database and conducted a cross-analysis of the collected drug targets and disease targets to construct a PPI network diagram. Furthermore, we collected normal and diseased gene data from UTI patients from the GEO database, conducted screening for DEGs, and plotted volcano plots and heatmaps to visually display changes in gene expression. We uploaded the drug targets, disease targets, DEGs, and modular genes into Venn diagram analysis, successfully screening out 5 hub genes. For these hub genes, we conducted GO analysis and KEGG pathway analysis and verified the results through molecular docking techniques. In in vivo experiments, we established an evaluation system for the therapeutic effect of KZMK granules on rats with an LPS-induced cystitis model. Through urine sediment leukocyte detection, bladder index assessment, HE pathological staining, immunohistochemical analysis, serum inflammatory factor detection, and Western blot experiments, we verified that KZMK granules exert their therapeutic effect on UTIs by inhibiting the activation of the NLRP3 inflammasome.

Based on Kazakh medical literature and clinical studies, it has been established that *Kezimuke* granules exhibit properties that reduce inflammation, enhance blood circulation, and alleviate symptoms associated with urinary tract infections. They are used for urinary tract infections, cystitis, pyelonephritis, nephrotic syndrome, prostate enlargement, and urinary obstruction, and can also be used as a systemic strengthening agent. Many researchers have previously demonstrated that this formula has good anti-inflammatory, antibacterial, and therapeutic effects on metabolic syndrome [13]. This research endeavor aimed to identify the active constituents, their corresponding targets, and the multi-faceted pharmacological mechanisms of KZMK in the treatment of UTIs Figure 1. This was accomplished through the integration of network pharmacology with bioinformatics methodologies. Network pharmacology offers a framework for constructing multi-component and multi-target models, enabling a comprehensive elucidation of the intricate interactions between active compounds and target proteins from a systematic, network-centric perspective. Furthermore, in order to further enhance the reliability of the research findings, we not only incorporated molecular docking studies as a verification method but also conducted studies on therapeutic effects using rats with a bladder inflammation model established in vivo as a supplementary validation tool. To our current understanding, this research constitutes a groundbreaking endeavor in investigating the therapeutic efficacy and underlying mechanisms of KZMK in addressing urinary tract infections, thereby furnishing experimental evidence that may pave the way for its future application and development.

## 2. Results

### 2.1. Identification of Serum Components of KZMK Extract

**Figure 2:**
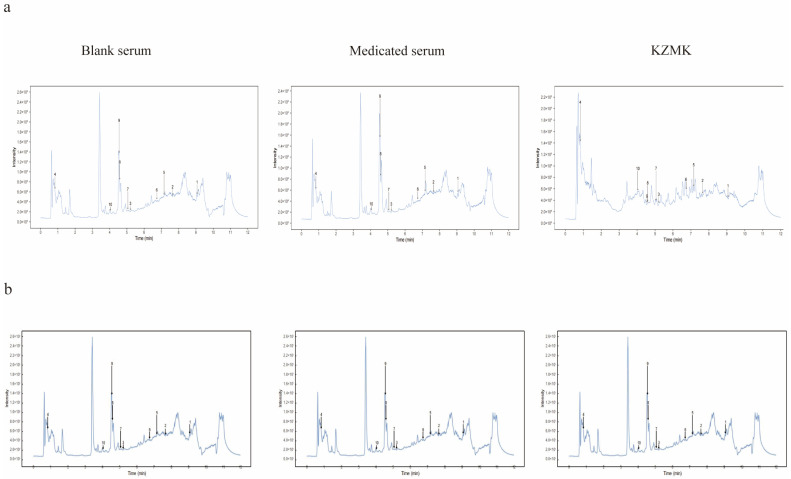
Identification of Serum Components Derived from KZMK Extracts. (**a**) Represents the TAIC in Positive Ion Mode for Diverse Serum Samples; (**b**) Illustrates the TAIC in Negative Ion Mode for Various Serum Samples.

The serum composition from KZMK was determined through a comprehensive LC-MS/MS analysis. The obtained LC-MS/MS spectra encompass those of the KZMK extract, as well as samples from the blank control and the KZMK-treated group, as depicted in Figure 2 for both negative and positive ion modes. Specifically, Figure 2a depicts the Total Anionic Ion Chromatogram (TAIC) in Positive Ion Mode for the various serum samples, while Figure 2b showcases the TAIC in Negative Ion Mode. Through this analysis, we were able to identify a comprehensive total of 17 serum components within the KZMK extract. Notably, four of these constituents were identified in negative ion mode, and an additional 13 components were identified in positive ion mode. These components are primarily constituted by flavonoids and triterpenoids, as detailed in Table 1.

### 2.2. Network Pharmacology Analysis

#### 2.2.1. Network Pharmacology Analysis

**Figure 3:**
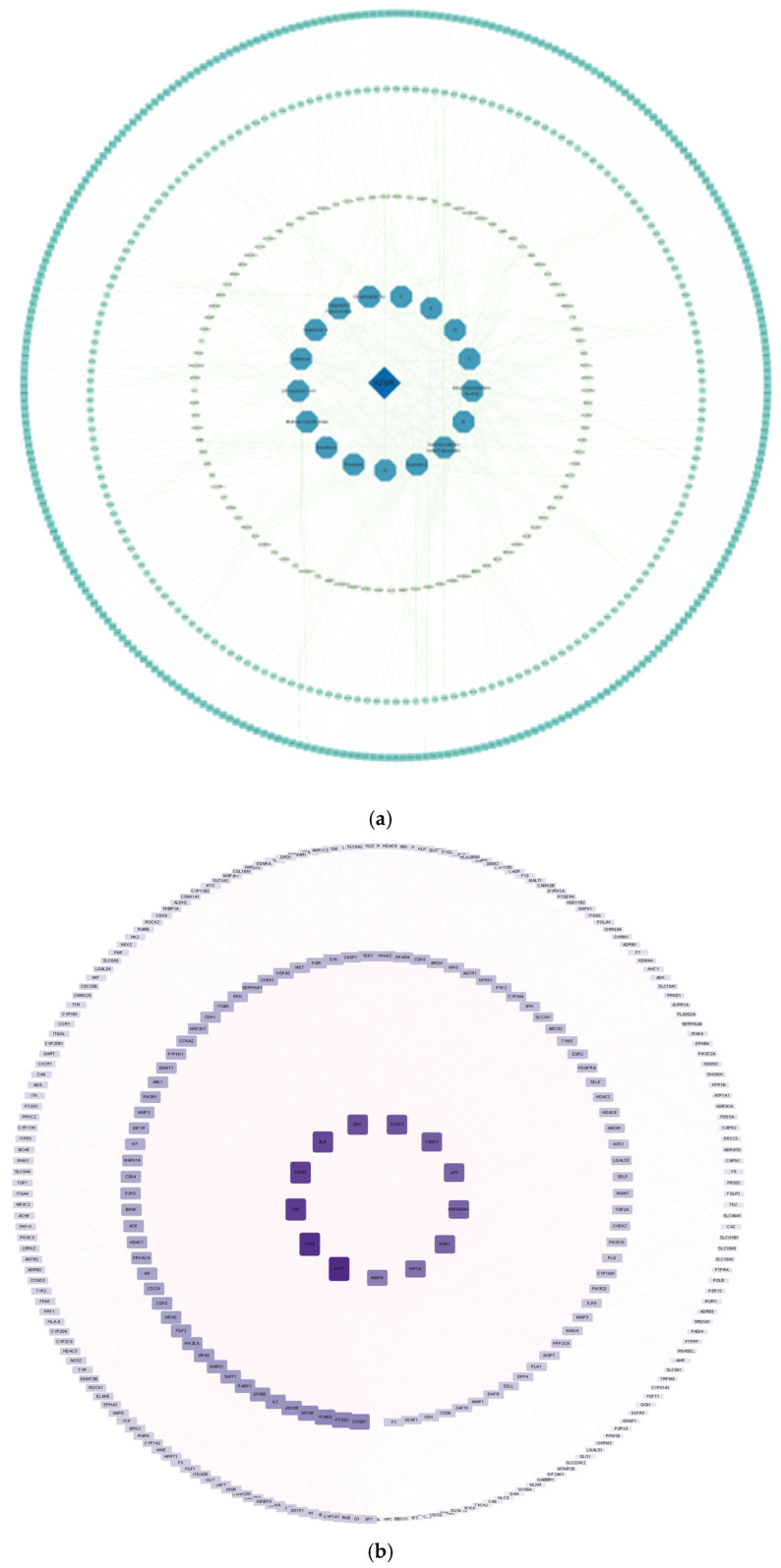

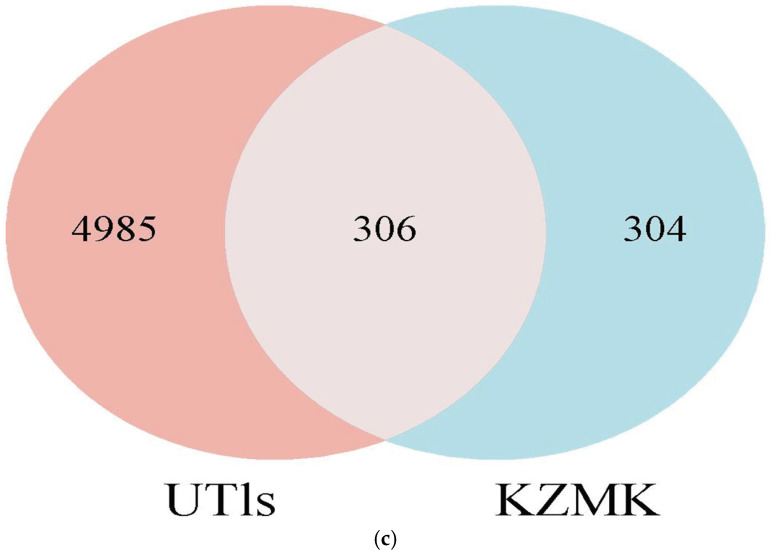
Network Pharmacology Analysis: From top to bottom, they are (**a**), (**b**), (**c**), respectively. (**a**) Ingredient-Target Network Diagram: The deep blue diamond core in the diagram represents KZMK. The surrounding blue hexagons represent the 17 serum ingredients of KZMK. Due to the excessive length of some component names, abbreviations such as ABCDEF have been used instead, with the specific distribution as follows. A: 5-hydroxy-6-methoxy-2-(4-methoxyphenyl)-7-[(2S,3R,4S,5S,6R)-3,4,5-trihydroxy-6-(hydroxymethyl)tetrahydropyran-2-yl]oxychroman-4-one; B: Glycyrrhizic acid; C: 7-hydroxy-2-(4-hydroxyphenyl)-8-[(2S,3R,4R,5S,6R)-3,4,5-trihydroxy-6-(hydroxymethyl) tetrahydropyran-2-yl] chroman-4-one; D: 5-hydroxy-6,7-dimethoxy-2-[4-[(2S,3R,4S,5S,6R)-3,4,5-trihydroxy-6-(hydroxymethyl) tetrahydropyran-2-yl]oxyphenyl]chroman-4-one; E: 3-[4,5-dihydroxy-6-(hydroxymethyl)-[(2S,3R,4R,5R,6S)-3,4,5-trihydroxy-6-methyltetrahydropyran-2-yl]oxy-tetrahydropyran-2-yl]oxy-5,7-dihydroxy-2-(4-hydroxyphenyl)chroman-4-one (Note: This ingredient is repeated with F, which may be a typographical error or data redundancy requiring further verification); F: 7-[(2S,3R,4S,5S,6R)-4,5-dihydroxy-6-(hydroxymethyl)-3-[(2S,3R,4R,5R,6S)-3,4,5-trihydroxy-6-methyltetrahydropyran-2-yl]oxytetrahydropyran-2-yl]oxy-3,5-dihydroxy-2-(4-hydroxyphenyl)chromen-4-one. The three outer circles of squares represent 610 targets, and the size of these squares visually reflects the node degree of the target proteins, i.e., how many ingredients they interact with. (**b**) Venn Diagram, (**c**) PPI Network Diagram: In this diagram, the size and color intensity of the squares are defined according to the DEGREE value.

Based on three traditional Chinese medicine ingredients, seventeen active drug compounds, and 610 targets, we constructed a comprehensive compound-target network utilizing Cytoscape version 3.9.1, as illustrated in Figure 3a. This intricate network comprises one primary drug, seventeen distinct compounds derived from it, and six hundred and ten corresponding compound targets. The compounds within this network were systematically organized based on their Degree values, where a higher Degree signifies a greater involvement in various biological functions, thereby indicating a stronger biological significance. Furthermore, we identified three hundred and six overlapping genes between the drug and the associated disease through the utilization of a Venn diagram, depicted in Figure 3b. Subsequently, we accessed the STRING database to acquire PPI information. This information was then integrated into Cytoscape 3.9.1 to generate a PPI network diagram, shown in Figure 3c. In this diagram, the size of each protein was adjusted according to its Degree value for enhanced visualization. 

#### 2.2.2. Acquisition of Differentially Expressed Genes

**Figure 4:**
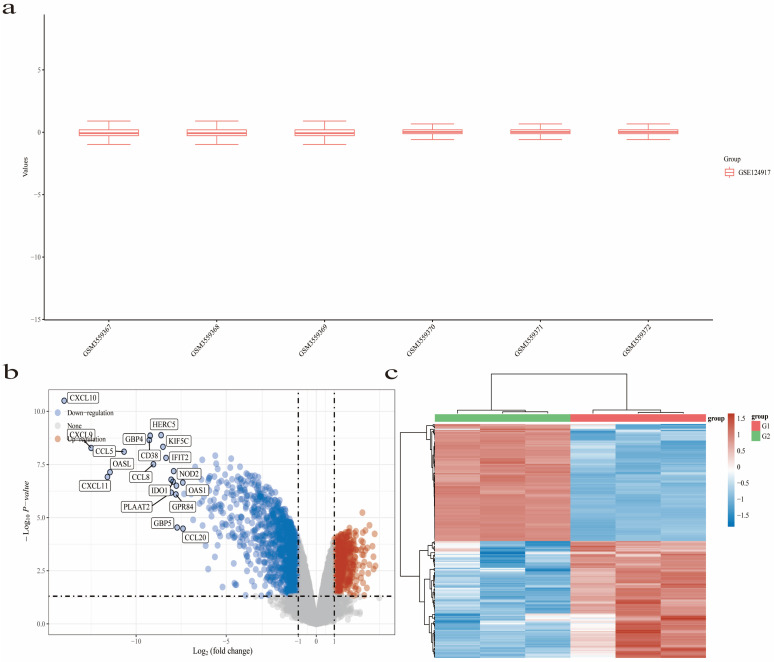
(**a**) presents the boxplot of the normalized data. In this visualization, various colors are utilized to distinguish between different datasets. The rows of the boxplot depict individual samples, while the columns represent the corresponding gene expression values within those samples. (**b**) depicts a volcano plot, constructed utilizing fold change values and adjusted *p*-values. Its purpose is to emphasize notable alterations in gene expression. Specifically, the volcano plot features red dots denoting upregulated genes; contrastingly, blue dots signify downregulated genes. The horizontal dotted line in the figure represents the significance threshold line. (**c**) presents a heatmap illustrating differential gene expression. Within this heatmap, a range of colors is utilized to depict the diverse trends of gene expression across various tissues. Notably, the heatmap highlights the top 50 genes with the most significant upregulation and downregulation, offering a comprehensive view of the most prominent changes in gene expression.

**Figure 5:**
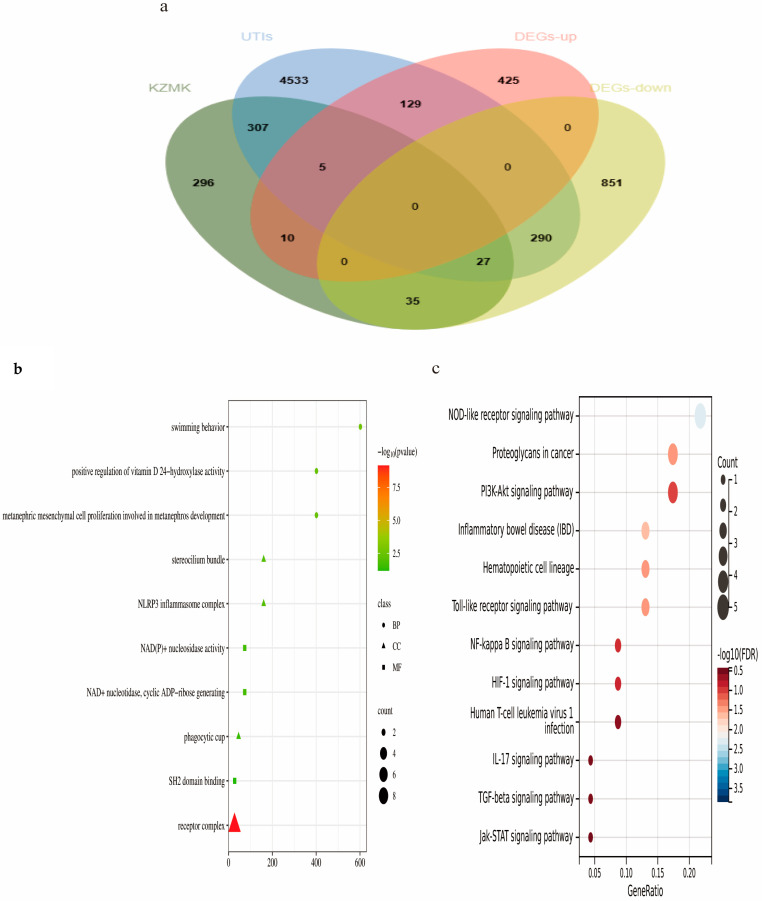
(**a**) Venn Diagram: Selection of Overlapping Genes. In this Venn diagram, green represents KZMK, blue represents UTls, while pink and yellow represent upregulated genes and downregulated genes, respectively; (**b**) GO Analysis; (**c**) KEGG Pathway Diagram.

We obtained information on UTI cellular models from the GEO database. Using R Studio (https://cran.rstudio.com/), we processed the data to identify differentially expressed genes (DEGs). Subsequently, we obtained a total of 1772 DEGs, comprising 569 upregulated genes and 1203 downregulated genes. To visualize the distribution of these DEGs, we generated two graphical representations: a volcano plot (Figure 4b) and a heatmap (Figure 4c). These figures provide insights into the pattern of gene expression alterations. Furthermore, we selected Caspase-1, NLRP3, STAT1, TLR4, and TNF as our focal genes, based on their significance as commonly implicated genes in association with various drugs and diseases, as well as exhibiting patterns of upregulation and downregulation (Figure 5a). 

#### 2.2.3. Functional Enrichment Analysis

To elucidate the underlying signaling pathways, we conducted GO analysis and KEGG pathway enrichment analysis utilizing the Cluster Profiler package within the R Bioconductor framework. The results of the GO analysis revealed that the overlapping genes, in terms of BP, were predominantly enriched in several critical areas: ventral midline development, the proliferation of metanephric mesenchymal cells involved in metanephros development, the enhancement of cerebellar granule cell precursor growth, and the promotion of the breakdown of protein-containing complexes. Furthermore, the CC terms included the NLRP3 inflammasome complex, phagocytic cup, caveola, and receptor complex. In terms of MF, these genes were enriched in nucleotidase activity, cyclic ADP-ribose generation, tumor necrosis factor receptor binding, transmembrane receptor activity, and protein tyrosine kinase activity (Figure 5b). Additionally, the results of the KEGG analysis revealed that the genes were predominantly concentrated in diverse pathways, such as Resistance to Antifolates, the NOD-like Receptor Signaling Pathway, Legionnaires’ Disease, Pertussis, the C-type Lectin Receptor Signaling Pathway, Inflammatory Bowel Disease, and Necroptosis. It is worth noting that, among these pathways, Resistance to Antifolates and the NOD-like Receptor Signaling Pathway (Figure 5c) exhibited the highest number of enriched targets, the greatest target enrichment values, and the lowest *p*-values, indicating their significance in the context of our study.

#### 2.2.4. Molecular Docking

**Figure 6:**
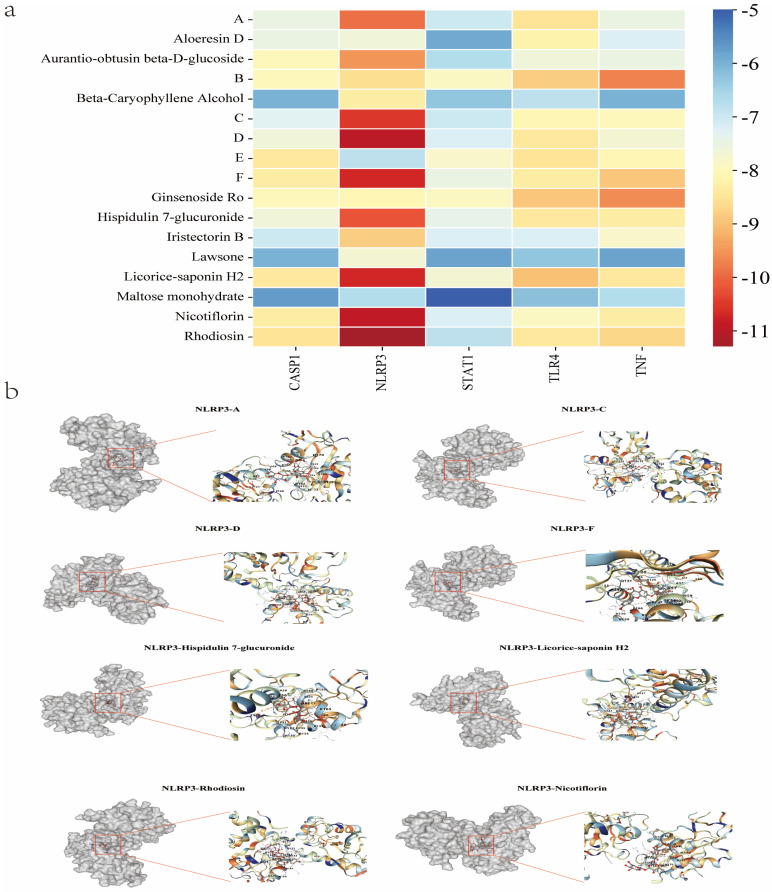
(**a**) A heatmap displaying the docking results of 17 serum components against 5 core targets for KZMK. The intensity of the colors in the diagram reflects the affinity or association between the receptor and the ligand, with darker colors indicating stronger binding activity. (**b**) The molecular docking results of NLRP3 with the following compounds are shown. The red box in the figure marks the location of the drug molecules.

Molecular docking was conducted utilizing the pivotal anti-UTl targets of KZMK, specifically Caspase-1 (PDB ID: IBMQ), NLRP3 (PDB ID: 6LG3), STAT1 (PDB ID: 3VNF), TLR4 (PDB ID: 4C7M), and TNF (PDB ID: 4V46), as receptors. Additionally, the seventeen serum components of KZMK served as ligands in this process. To assess the degree of compatibility between the constituents and the primary targets of KZMK, we evaluated the binding energy. Specifically, when both the ligand and receptor conformations stabilize, a lower energy value indicates a higher likelihood of binding. Generally speaking, a binding energy of ≤−4.25 Kcal/mol suggests the presence of some degree of binding between receptors and ligands. A binding energy of ≤−5.00 Kcal/mol indicates good binding, whereas ≤ −7.00 Kcal/mol signifies strong binding, Please refer to Table S1 for specific values. The results of this analysis are presented in Figure 6a. The findings revealed that NLRP3 exhibits strong binding energy with the components of KZMK. Further details are provided in Figure 6b.

### 2.3. White Blood Cell Count in Urine Sediment

**Figure 7:**
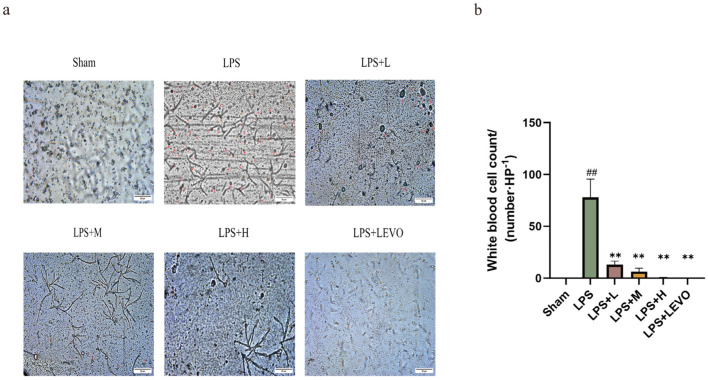
The impact of varying concentrations of KZMK on the WBC count in urine sediments was investigated. Representative photomicrographs were obtained to depict the WBC count in urine sediments (**a**). Additionally, a graphic representation of the WBC count was provided. The red arrow in the figure points to white blood cells. (**b**). The bar graph presented in (**b**) illustrates the mean and standard error of the mean (*n* = 6) for the WBC count. Statistical analysis revealed significant differences: ## *p* < 0.01 denote significant differences compared to the Sham group, respectively. Similarly, ** *p* < 0.01 indicates significant differences compared to the LPS group, respectively. Group descriptions are as follows: Sham represents the sham-operated group; LPS represents the lipopolysaccharide group; LPS + L represents the group receiving 0.676 g·kg^−1^·d^−1^ of KZMK after LPS instillation; LPS + M represents the group receiving 1.352 g·kg^−1^·d^−1^ of KZMK after LPS instillation; LPS + H represents the group receiving 2.703 g·kg^−1^·d^−1^ of KZMK after LPS instillation; LPS + LEVO represents the group receiving 10 mg·kg^−1^·d^−1^ of levofloxacin hydrochloride after LPS instillation.

As illustrated in Figure 7, the perfusion of LPS notably increased the quantity of WBCs in urine sediments, with a substantial jump from 0.0 ± 0/HP to 77.83 ± 7.268/HP (*p* < 0.01, *n* = 6). This elevation in WBC count was observed as a direct consequence of LPS perfusion. Furthermore, following a consecutive five-day oral administration of KZMK at dosages of 1.352 g·kg^−1^·d^−1^ and 2.703 g·kg^−1^·d^−1^, respectively, a significant decrease in the WBC count was observed (*p* < 0.01, *n* = 6). This reduction indicates the potential therapeutic effect of KZMK on LPS-induced inflammation in urine sediments. Additionally, as a positive control drug, LEVO at a dosage of 0.1 g·kg^−1^ also exhibited a significant reduction in the WBC count (*p* < 0.01, *n* = 6). The comparison with LEVO further validates the efficacy of KZMK in ameliorating the inflammatory response in urine sediments. 

### 2.4. Evaluation of Bladder Morphology and Bladder Index

**Figure 8:**
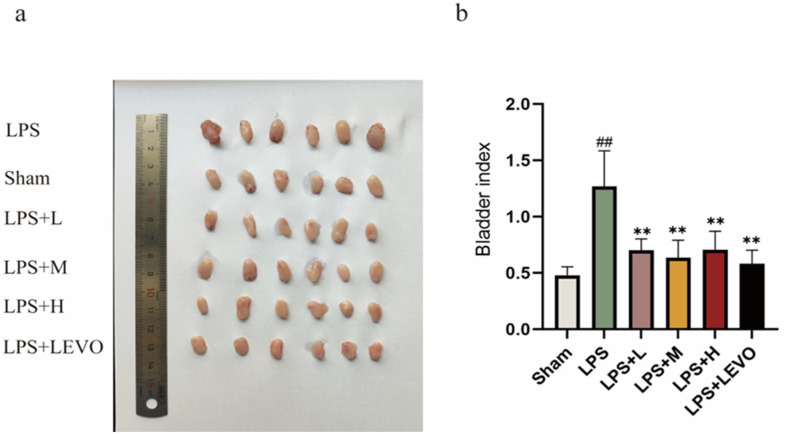
Influence: Effects of Different Concentrations of KZMK on Bladder Morphology and Bladder Index. The figure presents representative photographs of bladder morphology for each group (**a**) and a graphical representation of the bladder index (**b**). The bar graph presented in (**b**) illustrates the mean and standard error of the mean (*n* = 6) for the WBC count. Statistical analysis indicated significant variations: ## *p* < 0.01 represent significant differences in comparison to the Sham group, respectively. Likewise, ** *p* < 0.01 shows significant differences when compared to the LPS group, respectively. Group descriptions are as follows: Sham represents the sham-operated group; LPS represents the lipopolysaccharide group; LPS + L represents the group receiving 0.676 g·kg^−1^·d^−1^ of KZMK after LPS instillation; LPS + M represents the group receiving 1.352 g·kg^−1^·d^−1^ of KZMK after LPS instillation; LPS + H represents the group receiving 2.703 g·kg^−1^·d^−1^ of KZMK after LPS instillation; LPS + LEVO represents the group receiving 10 mg·kg^−1^·d^−1^ of levofloxacin hydrochloride after LPS instillation.

On the seventh day of the experiment, bladder tissues were collected for analysis. Upon examination, it was observed that the bladders of rats exposed to LPS exhibited enlargement when compared to those in the sham group. Additionally, the bladders of rats in the model group displayed noticeable congestion and edema. Consistent with these observations, the bladder indexes of the LPS-exposed rats increased significantly from 0.48 ± 0.07 to 1.27 ± 0.29 *(p* < 0.01, *n* = 6). Notably, when compared to the model group, both the medium-dose and high-dose treatment groups with KZMK, as well as the LEVO group, exhibited significant reductions in bladder congestion, edema, and bladder index (*p* < 0.01, *n* = 6). These findings are illustrated in Figure 8.

### 2.5. Bladder Histopathology Analysis

**Figure 9:**
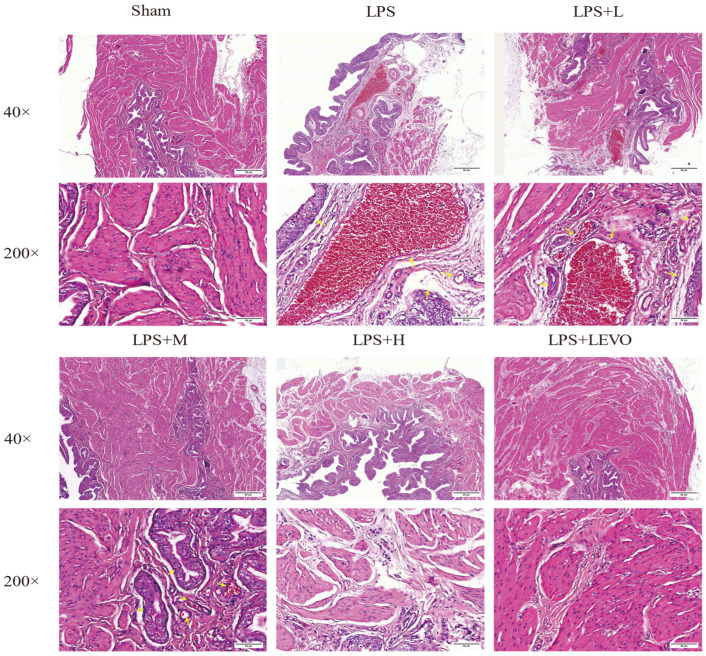
KZMK alleviates histopathological alte (magnification 40× and 200×). The yellow arrows respectively point to the areas where bleeding, edema, and severe immune infiltration occur. Group descriptions are as follows: Sham represents the sham-operated group; LPS represents the lipopolysaccharide group; LPS + L represents the group receiving 0.676 g·kg^−1^·d^−1^ of KZMK after LPS instillation; LPS + M represents the group receiving 1.352 g·kg^−1^·d^−1^ of KZMK after LPS instillation; LPS + H represents the group receiving 2.703 g·kg^−1^·d^−1^ of KZMK after LPS instillation; LPS + LEVO represents the group receiving 10 mg·kg^−1^·d^−1^ of levofloxacin hydrochloride after LPS instillation.

HE staining analysis revealed significant typical pathological features of cystitis in a rat model infused with LPS, specifically manifested as severe hemorrhage, edema, thickening in the submucosa, and infiltration and proliferation of inflammatory cells (Figure 9). In the low-dose KZMK treatment group, hemorrhage, edema, and inflammatory cell infiltration were still observed. However, when treated with medium-dose and high-dose KZMK, the bladder tissue damage was significantly improved. Meanwhile, the positive control drug levofloxacin (LEVO) also demonstrated an effect in reducing bladder damage. These findings collectively indicate that KZMK can effectively alleviate the histopathological damage to bladder tissue in LPS-induced cystitis rats in a dose-dependent manner.

### 2.6. Bladder Immunohistochemistry Analysis

**Figure 10:**
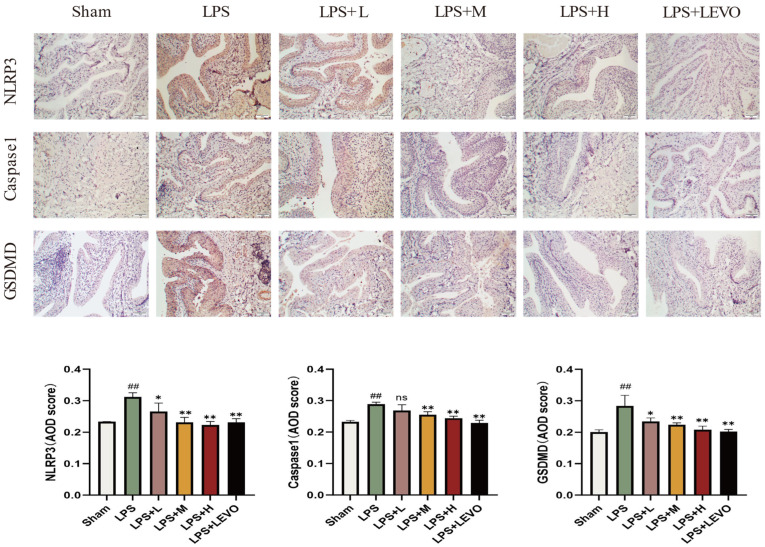
KZMK treatment attenuates the activation of NLRP3 inflammasomes in the bladders of LPS-instilled rats. Representative immunohistochemical images from bladder sections of rats in each group show staining for NLRP3, GSDMD, and Caspase-1. The scale bars for these photomicrographs are all 50 μm. In the statistical results illustrates the mean and standard error of the mean (*n* = 6) for the WBC count. Statistical analysis indicated significant variations: ns represents no statistical difference. ## *p* < 0.01 represent significant differences in comparison to the Sham group, respectively. Likewise, * *p* < 0.05 and ** *p* < 0.01 show significant differences when compared to the LPS group, respectively, and ns represents not statistically significant when comparing. Group descriptions are as follows: Sham represents the sham-operated group; LPS represents the lipopolysaccharide group; LPS + L represents the group receiving 0.676 g·kg^−1^·d^−1^ of KZMK after LPS instillation; LPS + M represents the group receiving 1.352 g·kg^−1^·d^−1^ of KZMK after LPS instillation; LPS + H represents the group receiving 2.703 g·kg^−1^·d^−1^ of KZMK after LPS instillation; LPS + LEVO represents the group receiving 10 mg·kg^−1^·d^−1^ of levofloxacin hydrochloride after LPS instillation.

The immunohistochemical staining outcomes depicted in Figure 10 revealed the presence of NLRP3, GSDMD, and Caspase-1 across all six groups of bladder tissues. Notably, the bladder tissues from the LPS group exhibited notably diminished expression levels of these proteins in contrast to those observed in the KZMK-administered group and the LEVO positive control group. To quantify the expression of NLRP3, GSDMD, and Caspase-1 in the bladder tissues, the average optical density (AOD) values were computed using ImageJ v1.8.0 software. The findings suggest that, in comparison to the LPS group, the KZMK-treated group demonstrated a dose-responsive reduction in the expression levels of NLRP3, GSDMD, and caspase-1 proteins within the bladder tissue. This decrease was statistically highly significant (*p* < 0.01). Furthermore, the positive control group’s drug also significantly downregulated the tissue expression of these proteins, resulting in a significant difference between the two groups (*p* < 0.05).

### 2.7. Serum Inflammatory Cytokines Assay

**Figure 11:**
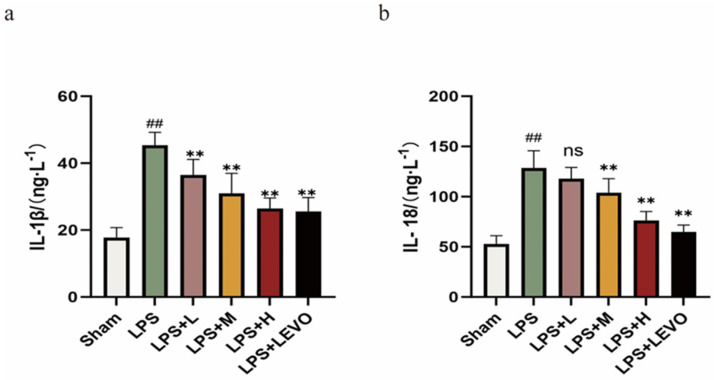
Displays the quantitative measurement results of IL-1β and IL-18 in the serum of rats from various groups. (**a**) represents the content of IL-1*β* in serum. (**b**) represents the content of IL-18 in serum. In the statistical results illustrates the mean and standard error of the mean (*n* = 6) for the WBC count. Statistical analysis indicated significant variations: ns represents no statistical difference. ## *p* < 0.01 represent significant differences in comparison to the Sham group, respectively. Likewise, ** *p* < 0.01 shows significant differences when compared to the LPS group, respectively, and ns represents not statistically significant when comparing. Group descriptions are as follows: Sham represents the sham-operated group; LPS represents the lipopolysaccharide group; LPS + L represents the group receiving 0.676 g·kg^−1^·d^−1^ of KZMK after LPS instillation; LPS + M represents the group receiving 1.352 g·kg^−1^·d^−1^ of KZMK after LPS instillation; LPS + H represents the group receiving 2.703 g·kg^−1^·d^−1^ of KZMK after LPS instillation; LPS + LEVO represents the group receiving 10 mg·kg^−1^·d^−1^ of levofloxacin hydrochloride after LPS instillation.

Compared to rats that received saline injections, the serum levels of IL-1β and IL-18 in the LPS group of rats exhibited a significant increase. KZMK demonstrated an ability to reduce serum IL-1*β* and IL-18 levels in a dose-dependent manner. Particularly in the high-dose treatment group, this reduction effect was particularly significant, with maximum reduction rates of up to 42% and 41% for IL-1β and IL-18, respectively (Figure 11).

### 2.8. Western Blot Analysis

**Figure 12:**
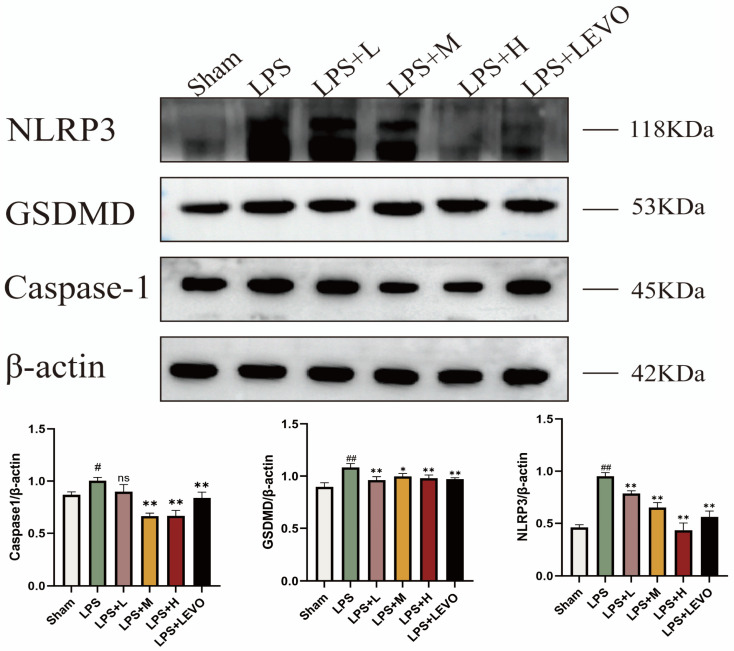
Displays the immunoblots and quantitative expression of representative proteins of the NLRP3 inflammasome in the bladder tissues of rats from various groups after treatment. Here, β-actin was used as the internal control standard. In the statistical results illustrates the mean and standard error of the mean (*n* = 6) for the WBC count. Statistical analysis indicated significant variations: ns represents no statistical difference. # *p* < 0.05 and ## *p* < 0.01 represent significant differences in comparison to the Sham group, respectively. Likewise, * *p* < 0.05 and ** *p* < 0.01 show significant differences when compared to the LPS group, respectively, and ns represents not statistically significant when comparing. Group descriptions are as follows: Sham represents the sham-operated group; LPS represents the lipopolysaccharide group; LPS + L represents the group receiving 0.676 g·kg^−1^·d^−1^ of KZMK after LPS instillation; LPS + M represents the group receiving 1.352 g·kg^−1^·d^−1^ of KZMK after LPS instillation; LPS + H represents the group receiving 2.703 g·kg^−1^·d^−1^ of KZMK after LPS instillation; LPS + LEVO represents the group receiving 10 mg·kg^−1^·d^−1^ of levofloxacin hydrochloride after LPS instillation.

Western blot analysis revealed protein expression levels revealed a significant upregulation trend of NLRP3, GSDMD, and Caspase-1 proteins in the bladder tissues of cystitis rats compared to the control group (*p* < 0.05), as shown in Figure 12. However, after treatment with KZMK extract, the expression levels of these proteins were significantly downregulated (*p* < 0.01). Specifically, the medium and high-dose groups of KZMK significantly inhibited the expression of NLRP3 and Caspase-1 proteins (*p* < 0.05), while the low-dose group did not achieve a statistically significant reduction in Caspase-1 protein expression. Additionally, all three different doses of KZMK downregulated the expression of GSDMD protein to varying degrees *p* < 0.05), with the low and high doses exhibiting particularly notable therapeutic effects.

## 3. Discussion

UTIs are common infections caused by bacteria, which can be acquired both in daily life and during medical procedures, thus becoming an important reason for the extensive use of antibiotics in the medical field [14,15]. LPS, an endotoxin produced by UPEC, acts as a potent activator of inflammatory responses and holds a central position as a crucial Pathogen-Associated Molecular Pattern (PAMP) in Gram-negative bacteria. Even at extremely low concentrations present in the bloodstream, it is sufficient to trigger intense inflammatory reactions. Therefore, this research project employs the method of intravesical injection of LPS to establish a rat model of cystitis, which serves as the subject of in-depth investigation [16].

The inflammasome constitutes a substantial protein complex tasked with regulating the processing of inflammation-associated proteins, including GSDMD and IL-1β, in response to cellular encounters with pathogens or non-infectious injuries [17,18,19]. Among them, the NLRP3 inflammasome is broadly expressed and susceptible to activation by diverse stimuli. Its activation occurs primarily through two mechanisms: firstly, the canonical pathway, necessitating two sequential signals the initial signal augments the expression of inflammation-related genes, whereas the subsequent signal initiates the assembly of the NLRP3 inflammasome [20,21,22]; secondly, via the TLR4 signaling pathway, specifically in human monocytes. Furthermore, a non-canonical route of NLRP3 inflammasome activation exists, triggered when caspase-11 in mice or its human equivalent, caspase-4, detects lipopolysaccharide within the cytoplasm [23,24]. *Kezimuke granules*, a classic prescription imbued with the wisdom of traditional Kazak medicine from Xinjiang, have demonstrated remarkable potential in treating a variety of diseases, such as urinary tract infections, cystitis, pyelonephritis, nephrotic syndrome, prostatic hypertrophy, and urination difficulties, as documented in Kazak medical literature and validated through clinical practice. However, despite its significant efficacy, scientific research on its specific mechanism of action remains relatively scarce. In response to this situation, this study employed multiple cutting-edge technologies to deepen our understanding.

Firstly, we utilized LC/MS/MS to accurately detect the blood components of KZMK. Subsequently, based on these blood components, we conducted in-depth network pharmacological and bioinformatics analyses to predict the potential hub genes and related signaling pathways involved. Building on this foundation, we innovatively established a cystitis model by injecting LPS into the bladders of rats, aiming to explore in detail the underlying mechanism by which KZMK effectively inhibits the triggering of the NLRP3 inflammasome. Thereby treating cystitis. This research provides a scientific basis for the modern application of this traditional medicine.

Chinese herbal medicines, due to their broad applicability, are highly favored in the treatment of various inflammatory conditions like urinary tract infections. However, the complexity of their constituents and targets poses significant challenges to in-depth exploration of their mechanisms of action [25]. In this study, LC-MS/MS technology was employed to identify a total of 17 components in the KZMK extract that can enter the bloodstream in their prototype form. Furthermore, network pharmacological analysis results indicate that among 610 targets, through the analysis of the constructed PPI network diagram, AKT1 and TP53 were found to be two proteins closely related to treatment. By analyzing GEO data, we screened out 1772 DEGs related to UTl. Through further target screening analysis involving drugs, diseases, and these DEGs, we identified five overlapping genes as core therapeutic genes, namely Caspase-1, NLRP3, STAT1, TLR4, and TNF. The results of the KEGG analysis revealed that these core genes are mainly enriched in pathways such as antifolate resistance, NOD-like receptor signaling pathway, Legionnaires’ disease, pertussis, C-type lectin receptor signaling pathway, inflammatory bowel disease, and necroptosis, with antifolate resistance and the NOD-like receptor signaling pathway being particularly prominent. Additionally, molecular docking results showed that these five core genes have good binding affinity with the 17 blood-borne components, among which NLRP3 exhibits superior binding affinity. Previous studies have found that among the components in the bloodstream of KZMK, Nicotiflorin and Ginsenoside can effectively inhibit the NF-*κ*B signaling pathway and reduce the activation of the NLRP3 inflammasome, thus demonstrating positive efficacy in disease treatment. However, research on the other components regarding the NLRP3 inflammasome and urinary tract infections is still lacking. In our subsequent research work, we will conduct a more in-depth exploration and analysis of the pharmacological activities of these components [26,27].

Urinary sediment analysis, as an economical and non-invasive diagnostic tool, holds significant importance for detecting bladder injury, and its positive results can provide strong support for the diagnosis of cystitis. Clinically, the WBC count in urinary sediments is a crucial indicator for assessing the urinary tract infection status [28,29]. Our research results indicate that, compared with the sham-operated group, the WBC count in the model group increased significantly, with a statistically significant difference (*p* < 0.01). Furthermore, compared with the model group, after treatment with medium and high doses of KZMK, the WBC count in the urinary sediments decreased significantly, with a statistically significant difference (*p* < 0.01). This result strongly confirms the anti-inflammatory efficacy of KZMK (Figure 7). Additionally, we observed that after LPS exposure, the bladder tissue exhibited obvious congestion and edema, accompanied by an elevated bladder index. However, after treatment with KZMK, these inflammatory injuries were significantly reversed, and the bladder index was also correspondingly reduced. The research results show that KZMK can effectively alleviate the symptoms of congestion and edema in bladder tissue in a dose-dependent manner (Figure 8).

Through histopathological examination methods, we can conduct an objective and precise quantitative assessment of the cystitis induced by lipopolysaccharide. This assessment system comprehensively covers three key morphological indicators of acute inflammation: edema, leukocyte infiltration, and hemorrhage [30]. In the rat model injected with LPS, the pathological features of cystitis are particularly prominent, specifically manifesting as severe hemorrhage, significant edema, notable thickening of the submucosa, and extensive infiltration of inflammatory cells (Figure 9). In the rat group treated with a low dose of KZMK, we can still observe hemorrhage, edema, and inflammatory cell infiltration. However, when medium and high doses of KZMK are used for treatment, the damage to the bladder tissue is significantly improved. Meanwhile, as a positive control drug, LEVO also demonstrates an effect in reducing bladder damage. These findings all strongly indicate that KZMK can effectively alleviate the histopathological damage to the bladder tissue of rats with LPS-induced cystitis in a dose-dependent manner.

The NLRP3 inflammasome, which functions as a fundamental complex, consists of three pivotal proteins: NLRP3, ASC, and the precursor form of caspase-1. The mechanisms underlying its activation encompass both the canonical and non-canonical signaling pathways [31]. In the canonical activation pathway, the initial trigger for NLRP3 inflammasome assembly is the activation of the TLR4/NF-κB signaling cascade, which subsequently stimulates pro-caspase-1 to convert IL-1 family members into their mature, active forms. In contrast, the non-canonical pathway is characterized by the pivotal role of Caspase-11, which not only independently fosters inflammatory cell death, IL-1α maturation, and secretion but also augments Caspase-1 activation, leading to increased production of IL-1β and IL-18. This study particularly examines the regulatory impact of KZMK on the non-canonical NLRP3 activation pathway. Upon NLRP3 activation, caspase-1 executes its proteolytic function, transforming pro-IL-1β and pro-IL-18 into biologically active cytokines and inducing GSDMD pore formation, ultimately facilitating the release of mature IL-1β and IL-18 into the extracellular milieu. This series of processes not only leads to pyroptosis of the cells but also strongly triggers an inflammatory response [32,33,34,35]. The immunohistochemical analysis unveiled that, in contrast to the sham-operated group, the model group exhibited notably heightened expression levels of NLRP3, Caspase-1, and GSDMD, with a statistically significant divergence between the two (*p* < 0.01). Further scrutiny revealed that, in comparison to the model group, both the LPS + M group and the LPS + H group demonstrated significantly decreased expression levels of NLRP3, Caspase-1, and GSDMD in bladder tissues (*p* < 0.01), as depicted in Figure 10. Moreover, the results from Western blot analysis corroborated the immunohistochemical findings, both indicating a dose-dependent reduction in the expression levels of NLRP3, Caspase-1, and GSDMD in the tissues of cystitis rats post-KZMK treatment, as illustrated in Figure 12. Additionally, we noted a significant decrease in the levels of serum inflammatory cytokines IL-1β and IL-18 following KZMK treatment, as shown in Figure 11. This result suggests that KZMK can effectively downregulate the levels of serum pro-inflammatory cytokines, thereby exerting a therapeutic effect against cystitis. In conclusion, the aforementioned results strongly confirm that KZMK granules effectively treat cystitis in rats by blocking the activation process of the NLRP3 inflammasome.

In summary, this study first examined the blood components and applied network pharmacology for prediction. Subsequently, we established an LPS-induced cystitis rat model and verified the anti-inflammatory effect of KZMK through in vivo experiments, thereby confirming the prediction results of network pharmacology. In subsequent in-depth research, we will utilize more types of in vivo, in vitro, or ex vivo models to further explore the exceptional performance of KZMK in preserving and ensuring the functional integrity of the bladder epithelial barrier, restoration plays a crucial role. At the same time, we will also combine chemical research to initiate monomer analysis, striving to uncover the key bioactive components of KZMK in the treatment of UTls. These research findings are expected to provide valuable clues and insights for discovering new drugs from natural products for the treatment of recurrent cystitis.

## 4. Materials and Methods

### 4.1. Reagents and Drugs

The three main constituent herbs are *Vaccinium myrtillus*, *Helichrysum arenium* (L.) Moech and *Equisetum hiemale* L. of KZMK particles were purchased from the local medicinal herb market in Altay region; Levofloxacin hydrochloride (LEVO) was purchased from Zhejiang Asia-Pacific Pharmaceutical Co., Ltd. (Shaoxing, China); BCA Protein Concentration Assay Kit (BL521A) from Tian gen Biotech Co., Ltd. (Beijing, China); Anti-GAPDH antibody (ab128915), Abcam; Anti-NLRP3 antibody (ab263899); Anti-GSDMD antibody (ab219800), Anti-Caspase-1 antibody (ab179515), Abcam (Cambridge, UK); IL-1β and IL-18 ELISA kits are offered by Jiangsu Fei Ya Brand (Nantong, China).

### 4.2. Animals

Female Sprague-Dawley rats, aged between 4 and 6 weeks, with an approximate body weight of 260 g ± 30 g, were acquired from the Animal Experimentation Center of Xinjiang Medical University. These animals were subsequently kept in an environment with controlled conditions of SPF grade, located within the confines of the center. The facility maintained a consistent temperature of 23 degrees Celsius, with a tolerance of plus or minus 2 degrees. In order to guarantee the rats’ adequate care and maintenance of their well-being, they were supplied with standardized feed and pure, sterile water for drinking. Furthermore, they were subjected to a consistent 12-h alternation of light and dark periods. It is crucial to mention that the conduct of this study was authorized by the Ethics Committee for Laboratory Animals at Xinjiang Medical University, with the ethics approval identifier being IACUC-JT20230423-20.

After a week of adaptation, the rats underwent a 12-h period in which their water intake was restricted. Then, they were anesthetized by intraperitoneal injection of pentobarbital sodium, with a dosage of 40 mg per kilogram of body weight. Once anesthesia was established, a catheter was inserted into the bladder through the urethra, reaching a depth of two to three centimeters. Following insertion, urine was noted to flow freely into the collection tube. To facilitate complete bladder emptying, a gentle pressure was applied to the abdominal region of the rats. The bladder was then filled with either 500 μL of 0.9% sterile saline or 500 μL of LPS (5 mg·mL^−1^). To make sure the bladder was completely filled with LPS or saline, the catheter was left in place for 30 min before being removed. Rats were randomly assigned to one of six groups on the first day following LPS/saline infusion: (1) LPS-induced cystitis rats (LPS), (2) LPS-induced cystitis rats (Sham), (3)–(5) LPS-induced cystitis rats receiving KZMK by gavage at low dose (0.676 g·kg^−1^·d^−1^), medium dose (1.352 g·kg^−1^·d^−1^), or high-dose (2.703 g·kg^−1^·d^−1^), and (6) LPS-induced cystitis rats receiving levofloxacin hydrochloride (10 mg·kg^−1^·d^−1^) by gavage. Following the infusion procedure, medications were given continuously for five days. The KZMK dosage of 1.352 g·kg^−1^·d^−1^ was the same as what was used in clinical settings. In order to gather 24-h urine for additional examination, the rats were put in metabolic cages on the sixth day. All animals were executed by cervical dislocation on the seventh day following the collection of blood from the ocular veins, and the bladders were taken for western blotting and histological examination.

### 4.3. Preparation of KZMK Granules

The formula consists of 115 g of *Vaccinium myrtillus* (bilberry), 384 g of *Helichrysum arenarium*, and 192 g of *Equisetum*. For these three ingredients, the *Helichrysum arenarium* and *Equisetum* plants undergo a two-stage extraction process. For each stage, ten volumes of 70% ethanol are utilized, employing the reflux extraction technique for a duration of one hour. Subsequent to the extraction stages, the resultant filtrates are meticulously combined. Following this combination, the ethanol is recovered through the application of reduced pressure. The filtered concentrate undergoes further processing to attain a clear, paste-like consistency, possessing a relative density within the range of 1.15 to 1.20 at 50 °C. This refined product is destined for diverse applications in the future. The *Vaccinium myrtillus* undergoes a triple decoction process, where each decoction employs ten volumes of water and lasts for one hour. Subsequent to these decoction stages, the resulting filtrates are meticulously merged. The merged filtrates are then concentrated under a vacuum until they attain a solid-to-liquid ratio of 1:5. Thereafter, the concentrated filtrate undergoes centrifugation at 4000 revolutions per minute for 15 min. The supernatant that is resultant from this centrifugation is further concentrated under a vacuum until it transforms into a transparent paste. This paste exhibits a relative density of 1.20 to 1.25 at 50 °C. This transparent paste is subsequently blended with another clear paste extracted using ethanol. After thorough mixing of the two pastes, the resultant mixture is dried under reduced pressure at a temperature of 60 °C. The dried mixture is subsequently pulverized and sieved to a particle size of 100 mesh. One part of the resultant dry extract powder is mixed with two parts of lactose. The mixture is then softened using 95% ethanol, preparing it for further use in various applications. The mixture is then granulated through a 16-mesh sieve, dried at 50 to 60 °C, and finally, size-adjusted to obtain the final granules. All the herbal ingredients in the prescription are provided by the Xinjiang Kazakh Medical Research Institute, and the *Kezimuke granules* are accurately prepared as dark brown coarse granules.

### 4.4. Preparation of Serum Samples for UPLC-MS/MS

The experiment was conducted by randomly assigning six rats to two distinct groups: a blank control group (comprising three rats, utilized for obtaining blank serum) and a KZMK treatment group (consisting of three rats, aimed at obtaining drug-laden serum). Specifically, the rats within the KZMK treatment group underwent gavage administration of a KZMK solution at a dosage of 1.352 g/kg·time, twice daily, over a span of three consecutive days. Unlike the experimental group, the rats in the blank control group received an equal volume of a carrier solution as a comparator. Prior to blood collection, these rats fasted for 12 h while having continuous access to water. After the last dose administration, blood was obtained from the orbital venous plexus of each rat. Subsequently, the blood samples were centrifuged at 3000 rpm for 10 min at 4 °C. A specific volume of 200 μL of the supernatant was then carefully aspirated and mixed with 800 μL of methanol. Following additional centrifugation at an elevated speed of 13,000 rpm for 10 more minutes, the supernatant was meticulously collected and dried with nitrogen gas. Next, the supernatant was redissolved in 100 μL of methanol. The redissolved supernatant underwent centrifugation once again at 13,000 rpm for 10 min. The resulting supernatant was then filtered through a 0.22 µm membrane filter to guarantee purity. The filtrate obtained from this process was utilized for the subsequent analytical procedures.

For the preparation of KZMK granules for analysis, a precise quantity of 100 milligrams of the sample was meticulously measured utilizing an analytical balance and subsequently placed into a 2.0 milliliter Eppendorf tube Servicebio (Wuhan, China). Subsequently, a 500 microliter aliquot of a pre-cooled extraction solvent, consisting of a methanol-to-water ratio of 4:1 (volume per volume), which also contained isotope-labeled internal standards, was introduced into the tube. The contents were then vigorously mixed for 30 s using a vortex mixer. To improve homogenization efficiency, two small steel balls were incorporated into the tube. Following this, the tube was homogenized in a device operating at 45 Hz for four minutes. Immediately after homogenization, the mixture was transferred to an ice-water bath to maintain a low temperature. Within this bath, the mixture underwent ultrasonic extraction for one hour. Subsequently, a static settlement phase was conducted, where the sample was placed in a refrigerator at −40 °C for an additional hour to further separate the components. In order to isolate the supernatant, the sample underwent centrifugation at a regulated temperature of 4 °C and a rotational velocity of 12,000 rpm. The centrifugal force, equivalent to 13,800× *g* at a radius of 8.6 cm, was maintained for a duration of 15 min. The resulting supernatant was then precisely filtered through a 0.22-micrometer membrane and transferred into a vial, preparing it for subsequent detection and analytical procedures.

### 4.5. UPLC-MS/MS Analysis

In this experimental study, we employed the Vanquish UHPLC (VN-S10-A-01) from Thermo Fisher Scientific (Waltham, MA, USA), equipped with a Phenomenex Kinetex C18 00A-4605-AN chromatographic column. The primary objective of this chromatographic column was to isolate the targeted compounds. The separation technique utilized two distinct mobile phases: specifically, Mobile Phase A, comprising an aqueous solution containing 0.01% acetic acid, and Mobile Phase B, a volumetric 1:1 mixture of isopropanol and acetonitrile. Additionally, the experimental setup included a sample tray maintained at a controlled temperature of 4 °C. During the experiment, a sample volume of 2 microliters was injected. For mass spectrometry analysis, we utilized the Orbitrap Exploris 120 (VN-S10-A-01) mass spectrometer controlled by Xcalibur4.4 software. This advanced instrument was capable of acquiring both first-order and second-order mass spectrometry data, offering comprehensive analytical perspectives. The mass spectrometer’s operational settings were meticulously configured as outlined below: the sheath gas flow was set to 50 arbitrary units, while the auxiliary gas flow was tuned to 15 arbitrary units. To guarantee effective sample vaporization, the capillary temperature was held constant at 320 degrees Celsius. Additionally, the mass spectrometer operated with a resolving power of 60,000 for the full mass spectrum and 15,000 for MS/MS analysis. The stepped normalized collision energy (SNCE) was varied at 20, 30, and 40 to achieve varying degrees of fragmentation. Depending on the sample’s ionization requirements, the spray voltage was adjusted to 3.8 kV for positive ion mode and −3.4 kV for negative ion mode, respectively.

### 4.6. Network Pharmacology

#### 4.6.1. Screening for KZMK and Urinary Tract Infections (UTIs) Targets

The standardized three-dimensional molecular structures, alternatively known as SMILES (Simplified Molecular-Input Line-Entry System) (https://pubchem.ncbi.nlm.nih.gov/ (accessed on 1 March 2024)) identifiers, of the obtained KZMK components were retrieved and sourced from the PubMed database. To identify and pinpoint the specific targets of these KZMK components, we harnessed the extensive resources available at the SIB Swiss Institute of Bioinformatics. In the subsequent step, we conducted a comprehensive search for targets that were associated with urinary tract infections. This extensive search was carried out by utilizing three reputable databases: the GeneCards Database, the OMIM (Online Mendelian Inheritance in Man) Database, and the DrugBank Database. Our search criteria were rigorously set to include only those targets that possessed a score of 20 or higher, thereby ensuring the relevance, significance, and importance of the identified targets.

#### 4.6.2. Network Construction

The compound-target interaction network was developed utilizing Cytoscape software, particularly its version 3.9.1. This specific version facilitated the smooth incorporation of active compounds and their corresponding targets pertinent to the drug of interest. Subsequently, we obtained PPI through the extensive utilization of the STRING database resources. Then, leveraging the PPIs obtained, we once again utilized Cytoscape version 3.9.1 to construct a meticulous protein-protein interaction network. This network provided a thorough visual depiction of the complex interactions among the proteins, enabling a detailed analysis and interpretation of the data.

#### 4.6.3. Differentially Expressed Genes Screening

Clinical information regarding UTIs was sourced from the GEO database, specifically dataset GSE124917 utilizing platform GPL21185. The retrieved data pertained to clinical samples. After a six-hour incubation period with a multiplicity of infection (MOI) set at 10, we assessed gene expression levels in two distinct cohorts. The first cohort comprised primary human renal fibroblasts induced by the urinary tract pathogen Escherichia coli strain CFT073. The second cohort served as a control, consisting of uninduced, healthy primary human renal fibroblasts. In order to ascertain genes that exhibit differential expression (DEGs), an analysis of differential expression was performed utilizing R Studio. The statistical significance was established according to the following benchmarks: an absolute log2 fold change exceeding 0.5 (|log2FC| > 0.5) and a *p*-value below 0.05 (*p* < 0.05).

#### 4.6.4. Functional Enrichment Analysis

Gene ontology analysis consists of three fundamental aspects: Biological Processes (BP), Molecular Functions (MF), and Cellular Components (CC). These components collectively aim to elucidate the functional attributes of genes [36]. To better characterize the hub genes discovered in our study, we utilized the Kyoto Encyclopedia of Genes and Genomes (KEGG) for enrichment analysis. This helped us to identify and explore the enrichment of diverse biological signaling pathways [37]. We subsequently applied the R programming language 4.3.0 and the Bioconductor ClusterProfiler package 4.4.4 for the analyses of gene ontology and KEGG enrichment. Our integration of the results from our first analyses within the R environment and execution of the ClusterProfiler package enabled us to perform enrichment analysis based on a gene-cluster-specific level for a more microscopic look. This procedure was intended to provide insight into the functional characteristics and interaction patterning of those identified hub genes for a more global appreciation of their roles [38].

#### 4.6.5. Molecular Docking

They used network pharmacology and bioinformatics to identify, through a comprehensive screening procedure, five key genes from the affected hubs—NLRP3, CASP1, STAT1, TLR4, and TNF. In order to reveal more insight into these targets, obtaining three-dimensional (3D) structures was one of the requirements. Thus, PDB [39] was accessed, and the 3D structures of these hub genes were downloaded in PDB format. Then, Titan and other proteins were prepared using AutoDock4.2.6 by removing water molecules and adding hydrogen atoms to ensure the better performance of docking simulations. The refined structures were saved in PDBQT format [40] for further analysis.

In the experiment for the detection of blood constituents, seventeen components were identified in the bloodstream and further explored for their structural properties. This was done using the TCMSP and PubChem databases for obtaining their corresponding proteins [41]. Then again, these proteins were subjected to refinement in AutoDock4.2.6, this time purifying from the water molecules before docking by treating with and removing H_2_O molecules and adding hydrogen atoms to determine accurate molecular representations. The refined ligand files were kept in the PDBQT format for the next analyses.

The structural files were first opened in the AutoDock4.2.6 software to allow for molecular docking, which is crucial in commencing the docking simulations. This allowed us to compute the minimum receptor-ligand binding energy and evaluate the receptivity of the interaction. On determining the binding energy, the PDBQT files would further be converted back into the PDB format using OpenBabel software 2.4.1 [42]. This was a necessary step that would help to interact with other software for subsequent research. Ultimately, further visualization and interpretation of the resultant molecular docking maps by means of the Python-based graphical front end PyMOL2.5 were conducted, which enabled us to provide the related significance underlying the complex interactions involved between the drug compounds and their hub genes. Thus, we might make inferences on possible binding modes and mechanisms.

### 4.7. In Vivo Experiments

#### 4.7.1. White Blood Cell Count in Urine Sediment

Urine sediments were processed and analyzed following the procedures detailed in reference [21]. In summary, a 24-h urine collection underwent centrifugation at 2500 rpm for 5 min. The supernatant was discarded, resulting in the retention of the urine sediment. This sediment was then resuspended in 200 μL of liquid. An aliquot of 20 μL from the resuspended sediment was aspirated and placed onto a microscope slide featuring an 18 mm × 18 mm cover slip. Utilizing the Leica DMi8 microscope, accurately determined the number of WBCs in the urine sediment and captured their images, providing strong support for subsequent in-depth analysis.

#### 4.7.2. Evaluation of Bladder Morphology and Bladder Index

On the seventh day following the infusion surgery, the rats were administered sodium pentobarbital to induce anesthesia. Immediately subsequent to anesthesia induction, the bladders of the rats were isolated and carefully weighed. To assess the degree of bladder inflammation, a comparative analysis was conducted. To evaluate the bladders, a comparison was made between their macroscopic appearance and the bladder index (BI). The BI was calculated by dividing the bladder weight by the body weight and expressing the result as (mg·g^−1^). In particular, this method facilitated a quantitative assessment that correlated the macroscopic features with the BI value.

#### 4.7.3. Bladder Histopathology and Immunohistochemistry Analysis

Tissue samples from rats’ bladders were fixed in 4% paraformaldehyde, dehydrated, and then embedded in wax blocks for easier sectioning. Hematoxylin and eosin-stained HE sections of tissue were microscopically analyzed. Before immunohistochemical analysis, the wax-embedded sections were dewaxed and treated with a solution of 3% hydrogen peroxidase to quench endogenous peroxidase activity. Next, blocking was done with 3% bovine serum albumin for 30 min, and after the removal of the blocking solution, the primary antibody was applied. Once the sections completely dried, a secondary antibody, horseradish peroxidase (HRP)-conjugated, was applied. Immunohistochemical staining was then examined under an optical microscope. Immunohistochemical analyses were conducted in duplicate on the samples to ensure accurate measurement and repeatability. For the expressiveness and quantified representation, images were taken and analyzed using semi-quantitative software under ImageJ, thereby providing the utmost precision and accuracy to the analysis.

#### 4.7.4. Serum Inflammatory Cytokines Assay

Blood samples were collected to isolate serum. Serum was obtained from blood using centrifugation at 3000 rpm for 20 min before sacrificing the rats. Concentrations of IL-1β and IL-18 in serum were then quantitated using the ELISA method. These measurements were made with the aid of ELISA kits designed for rats, and the entire assay was performed in strict accordance with the manufacturer’s instructions.

#### 4.7.5. Western Blot Analysis

Western blot analysis was undertaken following the manufacturer’s instructions regarding antibody conditions to detect proteins present in the bladder. Proteins were first isolated and separated by means of a 10% SDS-PAGE system. The separated proteins were subsequently transferred onto PVDF membranes for further investigations, followed by overnight shaking at approximately 4 °C after applying the specific primary antibodies onto the membranes: rat anti-GSDMD antibody diluted 1:1000, rat anti-caspase-1 antibody diluted 1:1000, rat anti-NLRP3 antibody diluted 1:1000, and rat anti-*β*-actin antibody diluted 1:2000. After overnight incubation, membranes were incubated with HRP-conjugated secondary antibodies diluted 1:5000 for 1 h. Ultimately, various methods were employed to visualize the proteins in the final analysis.

#### 4.7.6. Statistical Analysis

We carried out statistical analysis on the experimental results with GraphPad Prism 10.0.0 software. The data are presented in the form of mean values ± standard deviation. For two independent samples, we employed a *t*-test for statistical analysis. As for three or more samples, a one-way analysis of variance was used. A *p*-value less than 0.05 is considered to be statistically significant.

## 5. Conclusions

**Figure 13:**
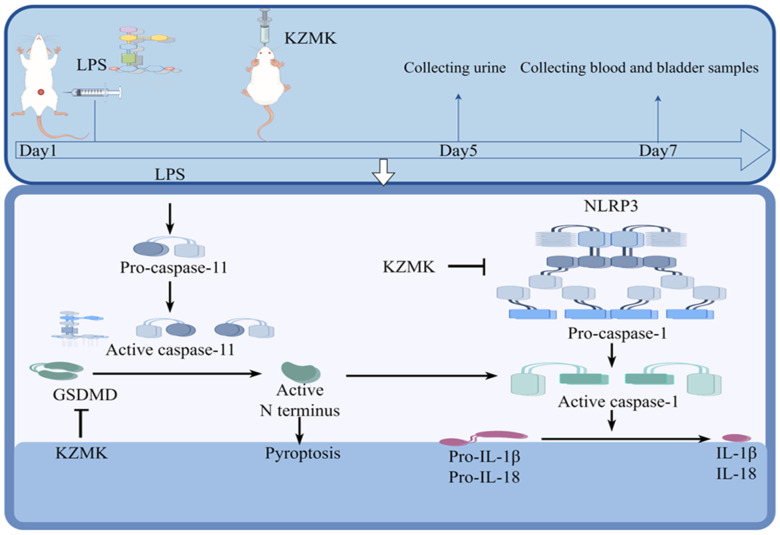
Illustrates the mechanism of action of KZMK in treating urinary tract infections by inhibiting the activation of NLRP3 inflammasome. Note: This diagram was created using Figdraw 2.0. In the figure, the arrows point to the downstream parts. The T-shaped arrows indicate blockage.

In this study, LC/MS/MS technology was employed to successfully detect 17 blood-borne components in the Kazakh medicine KZMK granules. Subsequently, we utilized network pharmacology and bioinformatics methods to predict five key hub genes involved in the treatment of UTIs with KZMK and further inferred its potential therapeutic targets. To confirm these forecasts, we undertook molecular docking studies and executed a battery of in vivo experiments. We established an animal model of cystitis by intravesically administering LPS, mimicking the clinical manifestations of infectious cystitis, to assess the therapeutic efficacy of KZMK in treating this condition. Our findings indicated that KZMK exhibited a potent capacity to mitigate bladder edema and inflammatory reactions elicited by LPS exposure. Additionally, it decreased the secretion of inflammatory cytokines and suppressed the activation of NLRP3 inflammasomes Figure 13.

## Figures and Tables

**Table 1:** The identified compounds of UPLC-MS/MS.

Compounds	Formula	Retention Time (min)	ION Mode	KZMK Extract	Blank Serum	Medicated Serum
Diammonium Glycyrrhizinate	C_42_H_62_O_16_	432′48″	Negative	18,766,070.02	0	30,073.55471
Nicotiflorin	C_27_H_30_O_15_	305′12″	Positive	16,350,147.23	0	15,289.76949
5-hydroxy-6-methoxy-2-(4-methoxyphenyl)-7-[(2S,3R,4S,5S,6R)-3,4,5-trihydroxy-6-(hydroxymethyl)tetrahydropyran-2-yl]oxy-chromen-4-one	C_23_H_24_O_11_	320′6″	Positive	76,995,735.65	0	72,415.57323
(2S,3S,4S,5R,6R)-6-[(2R,3R,4S,5S,6S)-2-[[(3S,6aR,6bS,8aS,12aR,14bS)-11-carboxy-4,4,6a,6b,8a,11,14b-heptamethyl-14-oxo-2,3,4a,5,6,7,8,9,10,12,12a,14a-dodecahydro-1H-picen-3-yl]oxy]-6-carboxy-4,5-dihydroxy-tetrahydropyran-3-yl]oxy-3,4,5-trihydroxy-tetrahydropyran-2-carboxylic acid	C_42_H_62_O_16_	432′48″	Negative	18,766,070.02	0	30,073.55471
Aloeresin D	C_29_H_32_O_11_	310′36″	Positive	19,794,295.13	0	17,004.26057
7-hydroxy-2-(4-hydroxyphenyl)-8-[(2S,3R,4R,5S,6R)-3,4,5-trihydroxy-6-(hydroxymethyl)tetrahydropyran-2-yl]chromen-4-one	C_21_H_20_O_9_	253′24″	Positive	51,257,590.5	0	39,272.54251
Rhodiosin	C_27_H_30_O_16_	295′30″	Positive	104,511,264.8	0	24,202.21409
Aurantio-obtusin beta-D-glucoside	C_23_H_24_O_12_	320′54″	Positive	27,694,119.77	0	36,460.56346
5-hydroxy-6,7-dimethoxy-2-[4-[(2S,3R,4S,5S,6R)-3,4,5-trihydroxy-6-(hydroxymethyl)tetrahydropyran-2-yl]oxyphenyl]chromen-4-one	C_23_H_24_O_11_	320′6″	Positive	76,995,735.65	0	72,415.57323
Maltose monohydrate	C_12_H_22_O_11_	61′18″	Negative	92,751,317.83	0	38,031.91904
3-[4,5-dihydroxy-6-(hydroxymethyl)-3-[(2S,3R,4R,5R,6S)-3,4,5-trihydroxy-6-methyl-tetrahydropyran-2-yl]oxy-tetrahydropyran-2-yl]oxy-5,7-dihydroxy-2-(4-hydroxyphenyl)chromen-4-one	C_27_H_30_O_15_	305′12″	Positive	16,350,147.23	0	15,289.76949
7-[(2S,3R,4S,5S,6R)-4,5-dihydroxy-6-(hydroxymethyl)-3-[(2S,3R,4R,5R,6S)-3,4,5-trihydroxy-6-methyl-tetrahydropyran-2-yl]oxy-tetrahydropyran-2-yl]oxy-3,5-dihydroxy-2-(4-hydroxyphenyl)chromen-4-one	C_27_H_30_O_15_	305′12″	Positive	16,350,147.23	0	15,289.76949
Iristectorin B	C_23_H_24_O_12_	320′54″	Positive	27,694,119.77	0	36,460.56346
Hispidulin 7-glucuronide	C_22_H_20_O_12_	319′00″	Positive	8,502,082.77	0	59,789.12333
Lawsone	C_10_H_6_O_3_	401′18″	Positive	14,586,267.49	0	11,889.90864
Ginsenoside Ro	C_48_H_76_O_19_	389′24″	Negative	21,575,462.8	0	152,947.9881
Lithospermic acid	C_27_H_22_O_12_	264′18″	Negative	245,512,707	0	36,592.9604
Beta-Caryophyllene Alcohol	C_15_H_26_O	251′48″	Positive	15,798,098.93	0	16,698.78599

The identified compounds of UHPLC-MS/MS.

## Data Availability

The datasets (GSE124917, GPL21185) for this study can be found in the Gene Expression Omnibus (GEO) database [https://www.ncbi.nlm.nih.gov/geo/ (accessed on (1 March 2024))]; Protein Data Bank (PDB) (https://www.rcsb.org/ (accessed on 15 April 2024)); PubMed database (https://www.ncbi.nlm.nih.gov/geo/ (accessed on 1 March 2024)); SIB Swiss Institute of Bioinformatics. (http://www.swisstargetprediction.ch/ (accessed on 20 January 2024)); GeneCards Database (https://www.genecards.org/ (accessed on 30 April 2024)); the OMIM Database (https://omim.org/ (accessed on 10 May 2024)); DrugBank Database (https://www.drugbank.com/ (accessed on 25 March 2024)); STRING database (https://cn.string-db.org/ (accessed on 12 February 2024)). All the data in this paper support the results of this study; other datasets used and/or analyzed during the current study are available from the corresponding author upon reasonable request.

## Supplementary Materials

The following supporting information can be downloaded at: https://www.mdpi.com/article/10.3390/ijms26041764/s1.
