# Supplementary material for: Bioinformatics Analysis of the Anti-Inflammatory Mechanism and Potential Therapeutic Efficacy of Kezimuke granules in Treating Urinary Tract Infections by Inhibiting NLRP3 Inflammasome Activation
Source: Int J Mol Sci. 2025 Feb 19;26(4):1764. doi: 10.3390/ijms26041764 (PMC11854959; doi:10.3390/ijms26041764)
Supplement: Supplementary file 1 [file ijms-26-01764-s001.zip › ijms-3440321-supplementary.pdf]

Table S1. Molecular docking binding energy results

Compounds	CASP1	NLRP3	STAT1	TLR4	TNF
A	-7.5	-9.9	-7	-8.5	-7.5
Aloeresin D	-7.5	-7.6	-5.9	-8.2	-7.2
Aurantio-obtusin beta-D-glucoside	-8	-9.5	-6.7	-7.6	-7.5
B	-8	-8.6	-7.9	-8.8	-9.7
Beta-Caryophyllene Alcohol	-6	-8.3	-6.3	-6.8	-6
C	-7.3	-10.5	-7	-8.1	-8
D	-7.6	-11	-7.2	-8.4	-7.7
E	-8.4	-6.8	-7.8	-8.5	-8.1
F	-8.3	-10.7	-7.5	-8.3	-8.9
Ginsenoside Ro	-8	-8.1	-7.9	-8.9	-9.6
Hispidulin 7-glucuronide	-7.6	-10.2	-7.4	-8.4	-8.3
Iristectorin B	-7	-8.8	-7.2	-7.2	-7.8
Lawsone	-6	-7.7	-5.8	-6.3	-5.8
Licorice-saponin H2	-8.4	-10.7	-7.7	-9	-8.4
Maltose monohydrate	-5.7	-6.7	-5	-6.2	-6.7
Nicotiflorin	-8.3	-10.9	-7.2	-7.9	-8.3
Rhodiosin	-8.5	-11.3	-6.8	-8.4	-8.7
